# Social circuits and their dysfunction in autism spectrum disorder

**DOI:** 10.1038/s41380-023-02201-0

**Published:** 2023-08-24

**Authors:** Masaaki Sato, Nobuhiro Nakai, Shuhei Fujima, Katrina Y. Choe, Toru Takumi

**Affiliations:** 1 Department of Neuropharmacology, Hokkaido University Graduate School of Medicine, Kita, Sapporo, 060-8638 Japan; 2 Department of Physiology and Cell Biology, Kobe University School of Medicine, Chuo, Kobe, 650-0017 Japan; 3 Department of Psychology, Neuroscience & Behaviour, McMaster University, Hamilton, ON Canada; 4 RIKEN Center for Biosystems Dynamics Research, Chuo, Kobe, 650-0047 Japan

**Keywords:** Neuroscience, Autism spectrum disorders

## Abstract

Social behaviors, how individuals act cooperatively and competitively with conspecifics, are widely seen across species. Rodents display various social behaviors, and many different behavioral paradigms have been used for investigating their neural circuit bases. Social behavior is highly vulnerable to brain network dysfunction caused by neurological and neuropsychiatric conditions such as autism spectrum disorders (ASDs). Studying mouse models of ASD provides a promising avenue toward elucidating mechanisms of abnormal social behavior and potential therapeutic targets for treatment. In this review, we outline recent progress and key findings on neural circuit mechanisms underlying social behavior, with particular emphasis on rodent studies that monitor and manipulate the activity of specific circuits using modern systems neuroscience approaches. Social behavior is mediated by a distributed brain-wide network among major cortical (e.g., medial prefrontal cortex (mPFC), anterior cingulate cortex, and insular cortex (IC)) and subcortical (e.g., nucleus accumbens, basolateral amygdala (BLA), and ventral tegmental area) structures, influenced by multiple neuromodulatory systems (e.g., oxytocin, dopamine, and serotonin). We particularly draw special attention to IC as a unique cortical area that mediates multisensory integration, encoding of ongoing social interaction, social decision-making, emotion, and empathy. Additionally, a synthesis of studies investigating ASD mouse models demonstrates that dysfunctions in mPFC-BLA circuitry and neuromodulation are prominent. Pharmacological rescues by local or systemic (e.g., oral) administration of various drugs have provided valuable clues for developing new therapeutic agents for ASD. Future efforts and technological advances will push forward the next frontiers in this field, such as the elucidation of brain-wide network activity and inter-brain neural dynamics during real and virtual social interactions, and the establishment of circuit-based therapy for disorders affecting social functions.

## Introduction

**Fig. 1:**
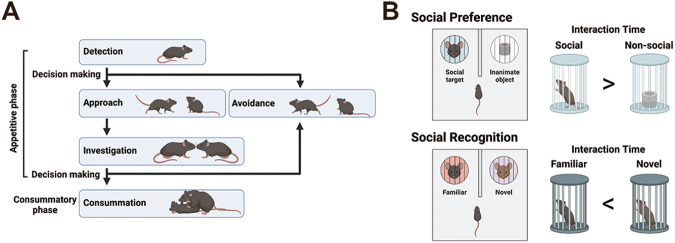
Social interaction of mice. **A** A simplified stream of mouse social interaction. **B** Social preference and social recognition. See main text for details. The figure was created using Biorender.com.

Individuals of certain organism species act cooperatively and competitively with peers for reproduction and survival. The ubiquity of such social behavior across various species, ranging primarily from insects to humans, suggests that it is evolutionarily advantageous. Social behavior includes various communications and interactions between two or more individuals of the same species [1]. When two conspecific individuals encounter each other, their interaction starts with an appetitive phase that involves detecting, approaching, and investigating the target individual as a social stimulus, followed by a consummatory phase that consists of stereotyped motor patterns that give rise to goal-directed actions such as aggression, mating, or parenting (Fig. 1A) [2, 3]. Although an aspect of social behavior is considered fundamentally innate and stereotypical, it is modulated by learning and memory to support its flexibility. Social behavior can also be categorized as affiliative or aggressive from the perspective of whether it has a positive and friendly nature or an intention to cause harm [4].

Social behavior is mediated by a distributed large-scale network of multiple brain structures. Expression of social behaviors is highly susceptible to dysfunction of this network, which is commonly observed in individuals with abnormal neurological and neuropsychiatric conditions such as stroke, schizophrenia, and autism spectrum disorder (ASD) [5–7]. ASDs are common early-onset neuropsychiatric conditions characterized by social communication deficits and restricted and repetitive patterns of sensory-motor behaviors. Although many non-genetic factors have been linked with ASDs, decades of research have established a strong causative relationship with genetics. Supporting its symptomatic heterogeneity manifesting as a broad spectrum, multiple types of genetic abnormalities have been associated with ASDs [8]. These include a large number of single genes, a major subset of which encodes synaptic molecules. In addition, multiple genetic abnormalities, including various copy number variants (CNVs) produced by deletion or duplication of chromosomal fragments [9], are heavily implicated in the pathogenesis of ASD. Due to the complex genetic nature of the disorder and the difficulty associated with studying how each (or groups of) genetic abnormalities contribute to ASD symptomology, investigating mouse models that mimic the genetic and clinical features of ASD thus provides a promising avenue toward elucidating mechanisms of abnormal social behavior and potential therapeutic targets for treating this disorder.

Studies have used many different behavioral paradigms to establish the social tendencies of rodents and the underlying neural circuit bases. For example, in an appetitive phase of social interaction, rodents show an inherent sociable tendency to prefer investigating a conspecific rather than an inanimate object, termed social preference (Fig. 1B). Rodents can also discriminate against each social target since they spend more time exploring novel individuals than familiar ones—an ability referred to as social recognition or social memory. Thus, investigation time decreases as they explore the same social target multiple times. Rodents also exhibit behavioral manifestations of empathy, such as observational pain, and prosocial behavior, such as consolation of stressed conspecifics [4, 10].

Here, we review recent progress and findings on neural circuit mechanisms underlying social behavior and circuit defects responsible for impaired social behavior in various ASD models, particularly with emphasis on rodent studies that use systems neuroscience approaches at the circuit level. Since excellent reviews on consummatory behaviors and their underlying mechanisms are already available [1–3], we primarily focus on the appetitive phase of social interaction in this review. We overview a set of brain networks that mediate social behaviors and their dysfunction in ASD model mice, specifically the neural circuits involving the medial prefrontal cortex (mPFC), the anterior cingulate cortex (ACC), the insular cortex (IC), and the neuromodulatory systems. Although an exhaustive listing of all relevant literature exceeds the scope of this review, we summarize some remarkable findings obtained from the recent studies of social behavior deficits of ASD mouse models in Table 1. Finally, we propose some major outstanding questions for future research in this field.

**Table 1:** Selected circuit-level studies of mouse models of ASDs.

ASD model	Gene or chromosome	Strain	Neural circuits	Social and circuit deficits	Rescue	References
Monogenic model	CAPS2	KO and OXT neuron-specific KO	OXT neurons in PVN	Impaired sociability Reduced OXT release from hypothalamus and pituitary	Intranasal OXT	Fujima et al., [135]
	CD38	KO	OXT neurons in PVN	Impaired social recognition memory Reduced OXT release from hypothalamus and pituitary	Subcutaneous OXT Restoration of CD38 expression in hypothalamus	Jin et al., [134]
	Cntnap2	KO	OXT neurons in PVN	Impaired sociability Fewer OXT neurons in PVN and reduced brain OXT levels	Intraperitoneal and intranasal OXT Pharmacological and chemogenetic enhancement of OXT release Postnatal intranasal OXT	Peñagarikano et al., [136]
		KO	OXT neurons in PVN	Impaired sociability Aberrant brain-wide functional connectivity	Intraperitoneal OXT Infusion of OXT receptor agonist into NAc Chemogenetic and optogenetic activation of OXT release	Choe et al., [142]
	Fmr1	KO and parvocellular OXT neuron-specific KO	OXT neurons in PVN	Impaired social reward learning		Lewis et al., [139]
	Nlgn3	KO	DA neurons in VTA	Impaired behavioral responses to social novelty Impaired OXT response in DA neurons in VTA Impaired translation in VTA	Oral administration of MAP kinase-interacting kinase inhibitor Restoration of Neuroligin 3 expression in DA neurons	Hörnberg et al., [140]
	Nf1	Heterozygous KO	mPFC-BLA	Impaired long-term social recognition memory Increased Mapk activation in PFC and BLA Aberrant network activity and impaired synaptic plasticity in BLA	Infusion of Pak1 inhibitor into BLA Genetic deletion of Pak1	Molosh et al., [36]
	Pten	Heterozygous KO	mPFC-BLA	Impaired social preference Hyperconnectivity of mPFC-BLA	Postnatal administration of S6K1 inhibitor Chemogenetic suppression of mPFC-BLA in adulthood	Huang et al., [35]
	Shank3	mPFC-BLA specific Δex4-9 KO	mPFC-BLA	Impaired sociability Hyperactivity and altered E/I balance in mPFC-BLA	Partial rescue by optogenetic inhibition of mPFC-BLA	Kim et al., [32]
		Heterozygous Δex21 KO	mPFC	Impaired social preference HDAC2 upregulation, reduced NMDAR function and F-actin in mPFC	Administration of HDAC inhibitor HDAC2 knockdown in mPFC	Qin et al., [38]
		Δex13-16 KO (Shank3B KO) and CRISPR/Cas9-mediated ACC-specific KO	ACC	Impaired sociability Excitatory synaptic dysfunction in ACC	Restoration of SHANK3 expression in ACC	Guo et al., [51]
		Δex13-16 KO (Shank3B KO)	OXT neurons in PVN	Impaired sociability Fewer OXT neurons in PVN and impaired OXT-dependent synaptic plasticity in VTA	Intranasal OXT Oral administration of *Lactobacillus reuteri*	Sgritta et al., [137]
		KO rat (premature stop codon in ex6)	HP-mPFC	Impaired long-term social recognition memory Impaired synaptic plasticity in HP-mPFC	Intraventricular OXT	Harony-Nicolas et al., [138]
		shRNA-mediated early postnatal downregulation in VTA	DA neurons in VTA	Impaired social preference Altered excitatory synaptic transmission and reduced DA neuron activity in VTA	Postnatal administration of mGluR1 positive allosteric modulator	Bariselli et al., [141]
CNV model	15q11-13	Duplication	5-HT neurons in DRN	Impaired sociability Reduced excitatory synaptic transmission and low glucose metabolism in DRN	Postnatal administration of SSRI	Nakai et al., [124]
	16p11.2	DRN-specific deletion	5-HT neurons in DRN	Impaired sociability Reduced 5-HT neuron activity in DRN	Pharmacological activation of 5-HT1B receptors in NAc	Walsh et al., [125]
		Heterozygous deletion	LC-MC	Delayed motor learning Reduced LC activity and increased ensemble activity in MC	Pharmacogenetic activation of NA neurons in LC	Yin et al., [130]
Inbred model		BTBR T+tf/J	IC	Weak inhibition and impaired maturation of multisensory integration in IC	Postnatal administration of benzodiazepine	Gogolla et al., [56]
Non-genetic model		C57BL/6J with MIA	mPFC-BLA	Impaired sociability Increased glutamatergic synaptic strength in mPFC-BLA		Li et al., [33]

*ACC* anterior cingulate cortex, *BLA* basolateral amygdala, *DA* dopamine, *DRN* dorsal raphe nucleus, *E/I* excitatory/inhibitory, *ex* exon, *HDAC* histone deacetylase, *HP* hippocampus, *IC* insular cortex, *KO* knockout, *LC* locus coeruleus, *MC* motor cortex, *mGluR1* metabotropic glutamate receptor 1, *MIA* maternal immune activation, *mPFC* medial prefrontal cortex, *NMDAR* N-methyl-D-aspartate-type glutamate receptors, *OXT* oxytocin, *NA* noradrenaline, *NAc* nucleus accumbens, *PVN* paraventricular nucleus of hypothalamus, *SSRI* selective serotonin reuptake inhibitor, *VTA* ventral tegmental area, *5-HT* serotonin.

## Cortical control of social behavior

### mPFC

#### Function in social behavior

Neurons in the mPFC form reciprocal networks with various subcortical regions such as the nucleus accumbens (NAc), amygdala, ventral tegmental area (VTA), and raphe nucleus [11], and crucially regulates cognition, emotion, and behavior [12, 13]. Although the involvement of the mPFC in social behaviors is well-established [14], the functional significance of the overall excitability of mPFC neurons in social behavior is not straightforward to understand. While the firing rate of some mPFC neurons increases when a mouse approaches a stranger [15], optogenetically-induced sustained elevation of mPFC pyramidal neuron excitability reduces social interaction behavior [16]. Consistently, distinct neuronal ensembles that are activated (ON ensembles) or suppressed (OFF ensembles) during social interaction carry information on social salience and novelty in the mPFC [17], suggesting that mPFC neurons that are implicated in social behavior are composed of heterogeneous populations.

**Fig. 2:**
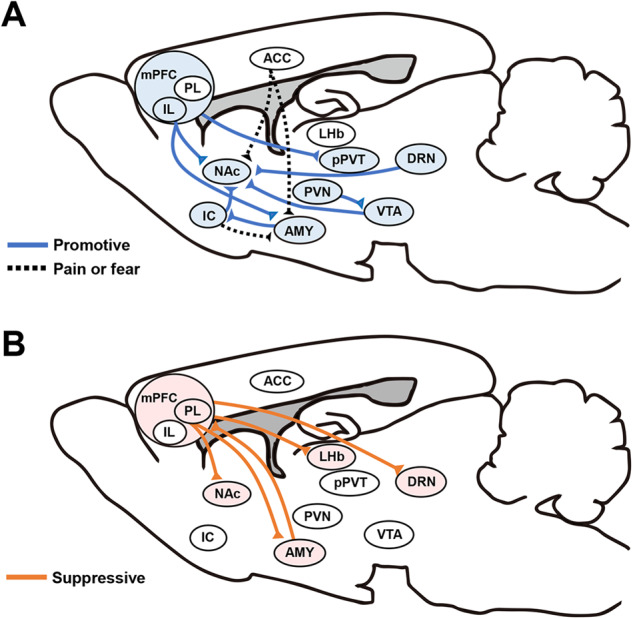
mPFC-centered neural circuits for control of social interaction. **A** Representative cortical and subcortical regions and their projections that promote social interaction (blue) and those that regulate pain and fear transfer (black dash). **B** Representative cortical and subcortical regions and their projections that suppress social interaction (red). See main text for details. ACC anterior cingulate cortex, AMY amygdala, DRN dorsal raphe nucleus, IC insular cortex, IL infralimbic area, LHb lateral habenula, mPFC medial prefrontal cortex, NAc nucleus accumbens, PL prelimbic area, pPVT posterior paraventricular thalamus, PVN paraventricular nucleus of hypothalamus, VTA ventral tegmental area.

Accumulating evidence indicates that mPFC controls social behaviors in a subregion- and projection target-specific manner (Fig. 2). Activation of projection terminals of the prelimbic (PL; an mPFC subregion) neurons at the NAc suppresses social preference [18]. During social interaction, the activity of basolateral amygdala (BLA)-projecting infralimbic (IL; another mPFC subregion) neurons is more prominent than BLA-projecting PL neurons, and inhibition of the IL-BLA pathway or activation of PL-BLA pathway reduces social behavior [19]. By contrast, a study that examined the role of opposite projections from the BLA to mPFC (including both PL and IL) demonstrated that activation of this pathway suppressed social interaction, whereas inhibition facilitated it [20], although this study did not discriminate between the BLA projections to the PL and IL. Another study showed that, during social interactions with a target mouse, dorsal mPFC–BLA coherence of local field potentials at the 4–7 Hz band is higher during leaving behavior compared to approach behavior [21], suggesting that rapid functional connectivity changes between the two areas accompany distinct aspects of social interaction.

The local circuit mechanisms regulated by the inhibitory interneurons also contribute to the intricate control of social behavior. Parvalbumin (PV)-positive interneurons target their axons to pyramidal neurons’ soma and suppress their excitability. Activation of PV-positive interneurons partially rescues a disruption in social preference elicited by elevated excitability of mPFC pyramidal neurons via normalizing the excitatory/inhibitory (E/I) balance [16]. Furthermore, social interaction increases low gamma oscillations and firing rates of PV-positive but not somatostatin (SST)-positive interneurons in the mPFC [22]. Conversely, inhibition of PV-positive but not SST-positive interneurons reduces low gamma power and impairs social interaction [22]. Surprisingly, however, activation of either PV-positive or SST-positive interneurons at low gamma frequency produces a prosocial effect [22], demonstrating the difference and commonality of two distinct classes of interneurons in local circuit control of social behavior.

Encounters with same-sex and opposite-sex conspecifics activate shared and distinct brain-wide networks [23], which can lead to different consummatory social behaviors such as fighting, mating, and parenting, according to the sex of the two individuals [1–3]. Sex-specific circuits and their activity thus mediate aspects of social behavior, and consequently, circuit dysfunction in ASD may also reflect such male-female circuit-level differences. In the mPFC of female mice, silencing a subpopulation of SST-positive interneurons that express oxytocin receptors (OTRs) resulted in the loss of social interest in male mice, specifically during estrus [24], demonstrating an example of local circuit control of sex-specific social behavior.

The mPFC also mediates adverse effects of social isolation stress. The activity of mPFC neurons that project to the posterior paraventricular thalamus (pPVT) is enhanced during social interaction, and suppression of this projection reduces sociability [25]. Interestingly, juvenile social isolation stress, known to induce sociability deficits in adulthood, impairs the activation of this pathway upon social exposure. Moreover, activating this pathway in adulthood rescues sociability deficits caused by juvenile isolation. Early social isolation also impairs social recognition through influence on the excitability of IL but not PL neurons [26]. Specifically, mice that experienced early social isolation lack the increased activity of NAc-projecting IL neurons during interaction with familiar conspecifics. Furthermore, inhibition of this pathway impairs social recognition without affecting social preference, and likewise, activation of this pathway rescues social recognition deficit in socially isolated mice [26]. Besides these circuits, recent whole-brain screening using rodent functional magnetic resonance imaging (fMRI) combined with chemogenetics identified lateral habenula (LHb) as a brain region that responds to PFC hyperactivity [27]. The role of LHb in regulating social behavior is demonstrated by the evidence that the activation of LHb neurons or PFC terminals in the LHb suppresses social preference. Since LHb is known to be involved in stress response and depression [28], elucidating whether this circuit also controls response to social stress is an important future question.

Despite the accepted importance of mPFC in controlling social behavior, many findings suggest that its distinct neural pathways are engaged in various contexts of social behavior. Thus, deciphering how the entire mPFC circuits operate during social interaction is still underway. A particular difficulty with this line of investigation arises from the complexity that mPFC also serves various non-social functions such as processing internal and external cues, decision-making, and flexible goal-directed behavior [14, 29]. The functional role of mPFC in the social domain is likely mediated by these general cognitive functions common to the non-social domain.

#### Implication in ASD model mice

As we saw earlier, the mPFC-BLA circuit is one of the important pathways for the control of social behavior [19, 30]. Several ASD mouse models exhibit abnormal social behavior and deficits in this circuit, suggesting that functional alterations in this pathway are crucially involved in social dysfunctions in ASD. SHANK genes encode a family of postsynaptic scaffold proteins associated with ASD [31]. Selective genetic deletion of *Shank3* in BLA-projecting mPFC neurons leads to elevated neural activity of this pathway and disrupts sociability [32]. The authors further show that similar PFC-BLA functional hyperconnectivity is also observed in individuals clinically diagnosed with ASD [32].

Maternal immune activation (MIA) and postnatal immune activation (PIA) by prenatal polyinosinic: polycytidylic acid-induced neuroinflammation in pregnant mice and early postnatal lipopolysaccharide injections, respectively, both result in social dysfunction and altered functions of mPFC-BLA pathway [33]. However, these two models have different cellular targets within this circuit. While MIA increases synaptic strength in glutamatergic mPFC projections to the BLA, PIA decreases feedforward GABAergic inhibitory postsynaptic responses in local BLA circuitry [33].

PTEN encodes a phosphatase that negatively regulates the PI3K-Akt-mTOR pathway, and germ-line heterozygous mutations of this gene are identified in individuals with ASD and macrocephaly [34]. PTEN heterozygous knockout mice display hyperconnectivity of mPFC to BLA projections, enhanced activity of mPFC and BLA in response to social stimuli, and social behavioral impairments [35]. The functional and behavioral abnormalities are reversed by pharmacological inhibition of S6 kinase beta-1 during development or by reducing the activity of the mPFC–BLA circuit in adulthood [35].

Neurofibromatosis type 1 (NF1) is a disorder that has a high prevalence of ASD, and mice lacking a single NF1 allele show deficits in long-term social learning and increased activation of mitogen-activated protein (MAP) kinase pathway in neurons from BLA and PFC [36]. These mice also display elevated GABA and glutamate neurotransmission and long-term potentiation in the BLA, and the social learning deficits are rescued by pharmacological blockade of p21 protein-activated kinase in the BLA [36].

Interplays between genetic and epigenetic mechanisms play an essential role in social functions and their deficits in ASD model mice [37]. A study has shown that Shank3 deficiency induces the histone deacetylase HDAC2 upregulation via a β-catenin–dependent mechanism, and its knockdown in the mPFC or treatment with the HDAC inhibitor romidepsin rescues the social deficits of heterozygous *Shank3*-deficient mice [38]. These findings underscore the likelihood of an epigenetic mechanism underlying social defects associated with Shank3 deficiency.

### ACC

#### Function in social behavior

The ACC in humans and rodents are highly interconnected with various brain areas primarily involved in emotional information processing, motivation, and autonomic function, such as orbitofrontal cortex, amygdala, NAc, hypothalamus, and autonomic brain stem nuclei [39]. The ACC has been implicated in various aspects of social cognition [40] – its activity is associated with other-referenced rewards [41] and negative valuations such as social anxiety [42, 43]. The ACC is also involved in the processing of the affective state of others [44]. Watching and learning are strategies to recognize cues for reward or punishment from others [45]. When observing another demonstrator mouse receiving an electric footshock, the observer mice show freezing behavior without experiencing the footshock, a process called observational fear learning [46]. Inactivation of ACC and an ACC-limited deletion of Cav1.2 calcium channel impaired this observational fear learning [47]. The ACC mediates these various types of social cognition processes via distinct projections. Recent studies demonstrate that the ACC-BLA circuit plays a crucial role in routing socially acquired aversive cue information and acquisition of observational fear conditioning [48] and that hippocampus-dependent 5–7 Hz oscillations in the ACC-BLA circuit in the right hemisphere are essential in empathic fear [49]. Furthermore, the ACC-NAc circuit regulates the social transfer of pain and analgesia, while the social transfer of fear depends on ACC-BLA circuit [50].

#### Implication in ASD model mice

Few studies have so far investigated the role of ACC in ASD model mice. Recently, a study has revealed morphological changes, hypoactivity, and weakened synaptic functions of pyramidal neurons in the ACC of mice deficient in *Shank3* [51], revealing this region’s possible implications in ASD neurobiology. The study further demonstrated that selective deletion of *Shank3* in the ACC resulted in synaptic dysfunction and social interaction deficits [51]. Moreover, optogenetic activation of ACC neurons, adult re-expression of *Shank3*, or pharmacological enhancement of α-amino-3-hydroxy-5-methyl-4-isoxazole propionic acid (AMPA)-type glutamate receptors improved social behavior in *Shank3* mutant mice [51]. Interestingly, activation of ACC pyramidal neurons in *Shank3* mutant mice rescued anxiety-like behavior. In contrast, their inhibition in wild-type mice did not affect it, indicating a possible dissociation of anxiety-like behavior and social dysfunction despite a proposed link between them [51].

### IC

#### Basic anatomy and function

**Fig. 3:**
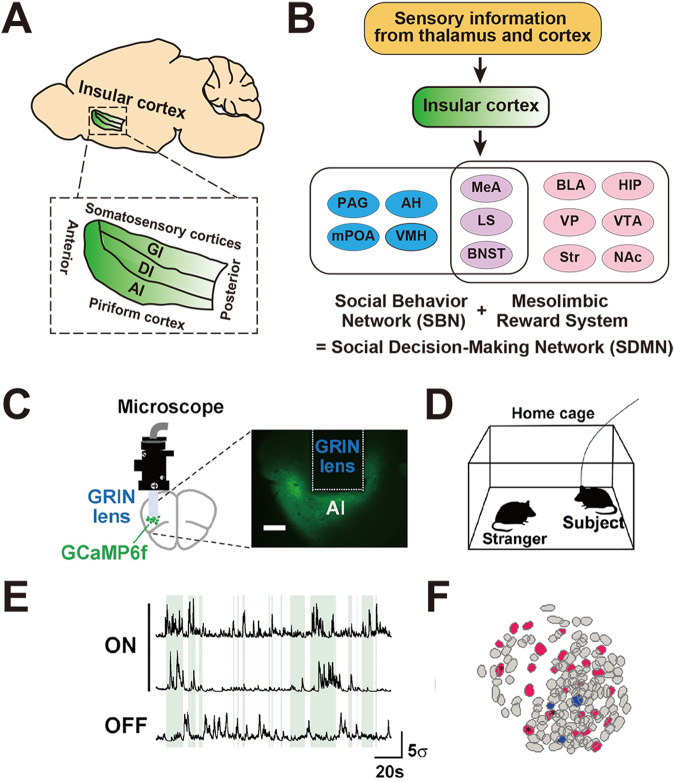
Insular cortex and social cells. **A** Anatomical organization of mouse IC. AI agranular IC, DI dysgranular IC, GI granular IC. **B** IC as a link between sensory inputs and social decision-making network. AH anterior hypothalamus, BLA basolateral amygdala, BNST bed nucleus stria terminalis, HIP hippocampus, LS lateral septum, MeA medial amygdala, mPOA medial preoptic area, NAc nucleus accumbens, PAG periaqueductal gray, Str striatum, VMH ventromedial hypothalamus, VP ventral pallidum, VTA ventral tegmental area. **C** Microendoscopic calcium imaging from the agranular IC (AI) in freely-moving mice. The activity of neurons labeled with the green fluorescent calcium indicator protein GCaMP6f is imaged using a miniaturized head-mounted fluorescence microscope through a chronically implanted GRIN lens. **D** Social interaction in a home cage. A male subject mouse with a microscope attached to its head is allowed to interact with a male conspecific stranger mouse in its home cage. **E** Example GCaMP fluorescence traces of social-ON cells and a social-OFF cell imaged during a home cage session. The periods of social interaction are indicated in green. **F** Anatomical distribution of social-ON cells (red) and social-OFF cells (blue) in a microendoscopic field of view. **C**–**F** are adapted from [75].

In this review, we draw particular attention to the IC as a unique cortical area that is still relatively understudied in the context of its involvement in the social behavior of rodents. The IC lies deep hidden below the frontal, parietal, and temporal lobes within the lateral sulcus in humans, and it is exposed mostly dorsal to the rhinal fissure and crossed by the middle cerebral artery on the lateral surface of the neocortex in rodents [52, 53]. Across species, the IC is subdivided along the rostrocaudal axis into two parts: the anterior insula and the posterior insula. In rodents, the IC is organized into three subdivisions arranged from dorsal to ventral and progressively devoid of the layer 4 granular layer: the granular subdivision, the dysgranular subdivision, and the agranular subdivision (Fig. 3A) [52, 54]. The IC forms an anatomic center with reciprocal connections to sensory, emotional, motivational, and cognitive systems, including the sensory and frontal cortices, amygdala, thalamus, and NAc, as well as with neuromodulatory inputs [55]. Rodent studies revealed that IC is involved in a wide variety of functions, including multisensory integration [56, 57], interoception [58, 59], pain [60, 61], taste [62–64], memory [65–67], emotion [58, 68], motivation [69], valence coding [70], physiological needs such as thirst and hunger [71, 72], aversive state processing [73, 74], and social functions such as social interaction [75, 76] and empathy [77–79]. In humans, the insula is also involved in self-awareness [80] and constitutes a part of the salient network that acts to detect novel and behaviorally relevant stimuli [81]. Atypical activation and connectivity of the IC are linked with various neuropsychiatric disorders such as schizophrenia and ASD [82, 83].

The anterior and posterior insulae are closely related by intensive intra-insular connectivity but differ in connectivity to other brain regions [84]. The posterior insula is thought to be a major site for receiving and processing visceral, gustatory, and other bodily and sensory signals and affective states, whereas the anterior insula may serve more like a higher association cortex that integrates the information from the posterior insula with top-down and valence signals to guide motivated behavior through its downstream targets such as the ventral striatum and the motor cortex [84].

Social behavior requires multiple steps of information processing where multimodal sensory signals are integrated with interoceptive and affective information to select an adaptive behavioral response. The IC is anatomically situated to influence the activity of a large-scale network called the social decision-making network (SDMN). The SDMN is proposed to be an evolutionarily conserved network of brain structures that regulates adaptive social behavior in response to salient environmental stimuli [85, 86]. The SDMN consists of the core nodes of the social behavior network (SBN), which includes lateral septum (LS), medial preoptic area, anterior hypothalamus, ventromedial hypothalamus, periaqueductal grey, medial amygdala (MeA), and bed nucleus stria terminalis (BNST) [87], and the mesolimbic reward system that includes NAc, VTA, ventral pallidum, BLA, hippocampus, LS, BNST, MeA, the last three of which are overlapping nodes with the SBN (Fig. 3B) [85, 88]. Each of the SBN nodes is reciprocally connected with all the others, is sensitive to gonadal steroids, and participates in more than one social behavior [87]. The mesolimbic reward system evaluates stimulus salience via dopaminergic signaling and thus mediates rewarding aspects of social interactions [85]. The SDMN can thus assess and respond to a social environment with adaptive behavioral decisions by interacting with these brain regions. The IC receives sensory inputs via direct thalamic and cortical afferents and is connected bidirectionally or unidirectionally with most nodes of the SDMN [84, 88]. Consistently, a network analysis of immediate early gene expression after social interactions with conspecifics demonstrates that the IC acts at the interface of “social” and “emotional” modules of the SDMN in the rat [77].

#### Function in social behavior

In line with the hypothesis that the IC links multimodal sensory processing and social decision-making, accumulating evidence broadly supports the idea that the IC is causally implicated in regulating social behavior. Pharmacological blockade of NR2B subunit-containing N-methyl-D-aspartate (NMDA)-type glutamate receptors in the agranular IC (aIC) decreased social investigation of male rats [89]. Activation of the pathways from posterior IC to central nucleus of the amygdala but not NAc interrupts ongoing social interaction, potentially due to behavioral inhibition by the anxiety signals mediated by this pathway [73]. Social stimuli activate vasoactive intestinal peptide (VIP)-expressing interneurons in anterior IC, and inhibition of this neuron subtype impairs social preference [76]. Moreover, a recent study uncovered that the IC directly participates in the coding of social exploration behavior [75]. In this study, microendoscopic calcium imaging visualized the activity of aIC neurons while the subject mouse interacted with a novel conspecific mouse in the home cage (Fig. 3C, D). This study identified two groups of aIC neurons that exhibited social interaction-related activity—a larger fraction of cells (social-ON cells) that were more active and a smaller fraction of cells (social-OFF cells) that were less active during social exploration (Fig. 3E, F). Interestingly, social-ON cells responded to mice regardless of their positions and consisted of multiple subsets of cells, each of which was preferentially active during exploration under a particular behavioral state (i.e., moving or stationary) or with a particular target of physical contact (i.e., nose, body, or anus). These findings suggest that neuronal ensembles in the aIC encode the ongoing status of social exploration at an individual cell level and the social salience of the interaction target at a population level. Elucidation of the projection targets and the function of insular social-ON and social-OFF cells is of particular interest and awaits future investigations.

The IC also plays a critical role in empathy. Empathy is the ability to perceive others’ emotional states and to understand others’ points of view [44]. It is common across many species, such as humans, apes, elephants, dolphins, and rodents [44], and is thought to be a major motivational drive for affiliative prosocial behaviors that benefit other individuals [4]. Empathy is impaired in various psychiatric, neurological, and neurodevelopmental conditions, including psychopathy and ASD [90]. While humans likely exhibit the most complex forms of prosocial behaviors, rodents also display a variety of behavioral manifestations of empathy, such as social modulation of pain sensitivity in mice [79, 91, 92], observational fear responses to footshocks to another mouse [47], allogrooming toward stressed conspecifics in prairie voles and mice [93–95] and helping of trapped conspecifics in rats [96, 97].

Recent studies have identified several key brain areas implicated in empathy [4, 44, 98]. The anterior IC, in conjunction with the ACC (see also above section on ACC), forms the most critical network nodes for both affective and cognitive aspects of empathy. In humans, the anterior IC is activated by both directly experienced and empathic pain [98, 99] and by feeling and observing disgust [100]. Patients with lesions in the anterior IC display deficits in empathic pain perception [101]. Moreover, individuals with ASD show behavioral deficits in inferring others’ social emotions and reduced activities in the right anterior IC [102]. In rodents, allogrooming towards stressed conspecifics increases c-Fos expression in OTR-expressing neurons in the IC in mice [94], and inactivation of the IC prevents socially-elicited hyperalgesia in mice [92]. Glutamatergic projection from the IC to BLA and synaptotagmin-2 and RIM3 in this pathway regulate observational pain [79]. In rats, silencing of the IC activity or blockade of insular OTRs prevented social approach and avoidance toward stressed conspecifics [77]. Moreover, inhibition of insular projections to NAc and BLA projections to posterior IC suppressed the social approach [78, 103], but global inhibition of the IC did not affect social novelty preference [78]. Inhibition of the anterior IC attenuates the helping of distressed conspecifics [97]. Taken together, these findings strongly indicate that both human and rodent ICs play a pivotal role in multiple forms of behavioral manifestations of empathy, ranging from social modulation of pain to helping behaviors.

#### Implication in ASD model mice

Investigation of IC dysfunction in ASD model mice is an understudied area of research. One notable study used intrinsic signal optical imaging to reveal impaired multisensory integration within the IC of BTBR T+tf/J inbred mice [56], a known model of idiopathic ASD that displays social interaction deficits compared to C57BL/6 strain [104]. The IC of these mice exhibited weak GABAergic inhibition and lacked enhanced responses to simultaneously presented audio-tactile stimuli. Early pharmacological enhancement of inhibition consistently rescued these deficits in adulthood, demonstrating the important role of proper E/I balance in the maturation of multisensory integration in the IC. Interestingly, multisensory integration deficits within the IC were also found in three separate monogenic ASD mouse models, specifically *Gad65*-, *Shank3*-, and *Mecp2*-deficient mice, strongly implicating this region as a site of shared pathophysiology across different types of ASD.

## Neuromodulatory control of social behavior

### Oxytocin, dopamine, and serotonin

#### Function in social behavior

The circuit mechanisms by which oxytocin regulates social behavior are attracting growing attention [105]. Oxytocin, a nine amino-acid neuromodulatory peptide, is mainly produced by a dedicated neuronal population in the paraventricular nucleus (PVN) and supraoptic nucleus of the hypothalamus [105]. These neurons send their axons to the posterior pituitary to release oxytocin into the periphery to promote uterine contraction and lactation. They also project centrally to a wide range of brain regions, such as the cerebral cortex, thalamus, amygdala, striatum, hippocampus, and midbrain [106, 107], to modulate various aspects of social and maternal behaviors.

In mice, genetic deletion of oxytocin or OTRs leads to abnormal social behaviors, including impaired social discrimination [108, 109]. Social recognition requires OTRs in the medial amygdala and hippocampus [110, 111]. Oxytocin enables pup retrieval behavior in female mice by enhancing pup call responses in the left auditory cortex [112]. In monogamous prairie voles, oxytocin plays a critical role in pair bonding [105]. An early study demonstrated that the pharmacological blockade of OTRs in NAc prevents mating-induced partner preference formation in this species [113]. Oxytocin release from PVN neurons onto dopaminergic neurons in the VTA and coordinated activity of oxytocin and serotonin in the NAc are required for the rewarding property of social interaction in mice [114, 115], demonstrating that interactions between multiple neuromodulatory systems play a significant role in controlling social behavior.

As discussed above, the NAc is critical in processing reward, motivation, and aversion and is deeply involved in social behavior. It receives dopaminergic inputs from the VTA and substantia nigra, and glutamatergic inputs from the amygdala, hippocampus, thalamus, and PFC [116]. Activation of VTA-NAc projections but not VTA-mPFC projections increases social interaction with a novel mouse [117]. Furthermore, dopamine signaling through D1 receptors, but not D2 receptors, is necessary for VTA stimulation-driven social behavior [117].

Mechanisms related to dopamine signaling in the NAc may contribute to adverse social stress-related mental illness. In rodents, social defeat stress (SDS), in which a subject receives a physical attack from an aggressor in an unavoidable environment, is often used as a model of stress-induced depression. A subpopulation of inbred C57BL/6 mice are susceptible to chronic SDS and exhibit depression-like behaviors such as low sociability and decreased locomotion after the stress, although the rest is resilient to this stress [118]. This phenotypic difference is controlled by firing patterns of the dopaminergic projections from VTA to NAc. Under SDS, the VTA dopamine neurons in susceptible mice show enhanced phasic firing patterns [118]. Optogenetic phasic activation of the VTA-NAc pathway induces a susceptible phenotype, while suppression induces resilience [119]. By contrast, inhibition of the VTA-mPFC pathway promotes susceptibility [119]. In the NAc, future resilient mice show increased baseline activity of D1 receptor-expressing medium spiny neurons before SDS and more significant social interaction-induced calcium transients compared to future susceptible mice [120]. On the other hand, in mPFC, the activation of D2 receptor-expressing subcortically-projecting pyramidal neurons disrupts normal social exploration behavior [121]. Dopamine signaling thus can regulate social behavior and stress responsiveness through distinct receptor subtypes in NAc and mPFC.

The serotonin system is also closely involved in social behavior. The dorsal and median raphe nuclei (DRN and MRN) are the core of serotonergic neurons. The rewarding properties of social interaction require serotonergic inputs from DRN and 5-HT1B receptors in the NAc [115]. In mice, the DRN GABA neurons receive excitatory inputs from the ventromedial PFC (vmPFC) [122]. The vmPFC-DRN pathway bidirectionally modulates socio-affective choices in the SDS paradigm [122], possibly by top-down modulation of GABA-mediated gating of the serotonergic output. In IC, infusions of antagonists of 5-HT1A serotonergic or D1/D5 dopaminergic receptors impaired the consolidation of social recognition memory in rats [123]. Importantly, dysfunction of the serotonin system is related to neurodevelopmental disorders such as ASDs [124, 125]. This point is discussed in detail in the next subsection.

#### Implication in ASD model mice

Human chromosome region 15q11–13 has five common breakpoints (BPs) that give rise to different CNVs, and the duplications of the imprinted region between BP2 and BP3 cause the most common and penetrant forms of ASD [9]. Specifically, while maternal duplications of this imprinted region are well-recognized risk factors for ASD, less frequent paternal duplications also increase the risk for ASD with a penetrance of ~20% [126]. Mice that genetically mimic human 15q duplication syndrome by paternal duplication of the 6.3-Mb syntenic region of mouse chromosome 7 display ASD-like behavioral abnormalities, including impaired social interaction [127]. These mice show decreased serotonin levels in several brain regions during postnatal development [128] and reduced excitatory synaptic drive and glucose metabolism in the adult DRN [124]. Restoration of serotonin levels by postnatal administration of the selective serotonin reuptake inhibitor normalizes the social behavior of these mice later in adulthood [124]. The effectiveness of early pharmacological serotonergic intervention in these mice is in accordance with the fact that ASD is an early-onset neurodevelopmental disorder and that serotonin plays a wide variety of roles in brain development before it acts as a neurotransmitter in the mature brain [6]. It should be noted that intervention strategies targeting different mechanisms have been successful in other mouse models of ASD (see review [129]). For example, in *Shank3* mutant mice discussed earlier [51], adult re-expression of Shank3 in ACC can improve social behavior. Combined, these findings demonstrate that social deficits in ASD model mice can be normalized by appropriate pharmacological and genetic interventions either during development or adulthood, depending on whether the relevant molecules are involved in neural circuit development or mature synaptic function.

Genetic deletion of the chromosome region syntenic to human 16p11.2 in dorsal raphe serotonergic neurons displays social behavioral deficits and decreases in their neuronal activity [125]. These sociability deficits are rescued by activating serotonergic signaling, especially 5-HT1B receptors in the NAc. In 16p11.2-deletion mice, pharmacogenetic activation of locus coeruleus noradrenergic neurons is sufficient to rescue delayed motor learning [130].

Multiple lines of evidence suggest a possible relationship between oxytocin and social impairment in ASD. For example, children with ASD show lower plasma oxytocin levels [131]. A genetic variation of the OTR relates to the levels of empathy [132], and the OTR gene and its single-nucleotide polymorphisms (SNPs) are associated with ASD [133]. A double-blind placebo-controlled crossover trial demonstrates that intranasal administration of oxytocin restores the activity of the anterior IC and enhances the ability to understand others’ social emotions in individuals with ASD [102]. Mice lacking CD38 or CAPS2 display abnormal social behavior, reduced plasma oxytocin levels, and deficits in oxytocin release from the hypothalamus and pituitary [134, 135]. Importantly from a therapeutic perspective, intranasal administration of oxytocin to a wide variety of ASD mouse models, including *Cntnap2*-deficient mice, *Shank3b*-deficient mice, prenatal valproic acid exposure model mice, BTBR T+ Itpr3tf/J inbred mice, and CAPS2-deficient mice, rescues social behavioral deficits [135–137] and intraventricular injection of oxytocin improves impaired social memory in *Shank3*-deficient rats [138].

Using mouse models, several recent investigations have revealed potential mechanisms that link oxytocin with the social phenotype of ASD. Oral administration of the bacterial species *Lactobacillus reuteri* corrects the PVN oxytocin levels and rescues their deficits in synaptic plasticity in the social reward circuits involving the VTA and social behavior in a vagus nerve-dependent manner [137]. Pathway-specific knockdown of FMR1, the gene disrupted in fragile X syndrome, in PVN parvocellular oxytocinergic neurons projecting to the NAc impairs social reward learning [139]. Mice bearing an autism-associated mutation in the synaptic adhesion molecule gene *Nlgn3* exhibit impaired oxytocin signaling in VTA dopaminergic neurons, disrupted translational regulation in the VTA, and altered behavioral responses to social novelty [140]. An orally-administered inhibitor of MAP kinase-interacting kinases rescues translation and restores oxytocin signaling and social novelty responses in these mice [140]. An shRNA-mediated early postnatal downregulation of *Shank3* in mouse VTA alters excitatory synaptic transmission, including AMPAR/NMDAR ratio and activity of dopaminergic neurons, resulting in deficits in social preference. Treatment with a positive allosteric modulator of mGluR1 during early life normalizes the AMPAR/NMDAR ratio and reverses social deficits in adulthood [141]. Recent work [142] has identified a mechanism by which oxytocin enhances sociability of *Cntnap2*-deficient mice. This study used a combination of mouse fMRI and c-Fos-iDISCO+ activity mapping [143] as a robust way to identify aberrant brain network activity associated with social deficits in *Cntnap2* knockout mice. Exogenously administered oxytocin strongly activates several SDMN regions (see above section on IC and SDMN) and normalizes aberrant brain network activity [142]. Moreover, chemogenetic stimulation of endogenous oxytocin release strongly activates the NAc and rescues social deficits [142]. Remarkably, restoring endogenous oxytocin signaling specifically in the NAc shell (NAcSh), an NAc subregion associated with social learning and behavior [144, 145], is sufficient to increase social interactions in these mice [142]. These results provide a link between an ASD-linked gene mutation and impaired oxytocin modulation of the SDMN as a potential mechanism underlying the social phenotype of ASD.

## Conclusions and future outlook

We have discussed recent findings that have elucidated critical brain circuits governing different aspects of social behavior. Studies using modern neuroscience tools that monitor and manipulate circuit activity have significantly advanced our knowledge of their function and dysfunction in normal mice and various ASD models toward a goal of a deeper understanding of human social behavior and relevant disorders. Social behavior is mediated by a distributed brain-wide network among cortical (e.g., mPFC, ACC, IC) and subcortical (e.g., NAc, BLA) structures and neuromodulatory systems (e.g., oxytocin, dopamine, serotonin). We drew particular attention to the IC as a unique cortical area in this review as it plays a vital role in multisensory processing, monitoring of social interaction, social decision-making, and empathy. Studies of ASD mouse models have shown that dysfunctions in mPFC-BLA circuitry and neuromodulatory systems are prominent. Pharmacological rescues by local or oral administration of various drugs have provided valuable clues for developing new therapeutic agents for ASD. In the future, technical advances that enable more precise tracing, recording, and manipulation of specific circuits and the introduction of new social behavioral assays will allow us to tackle important conceptual issues and make subsequent groundbreaking discoveries in this field. We here outline some major open questions regarding social behavioral mechanisms and developing potential treatments for ASD.

1. Each brain region, such as the mPFC, is connected with functionally diverse target regions. How does a large-scale brain-wide network operate while mice engage in social behavior? Elucidation of this problem will give us an important insight into near-whole-brain level biological principles of social behavioral mechanisms beyond a single circuit level. Besides investigating interactions between two or more defined circuits, an interplay between multiple neuromodulatory systems is another crucial point of interest. Analyses of network-level compensatory mechanisms elicited by primary circuit defects in ASD models will also substantially deepen our understanding of the dysfunction of autistic brains. Recently, simultaneous multi-site electrical recording from multiple brain regions has identified a brain-wide network that encodes individual rewarding social experiences [146]. Optical methods that allow parallel recording from multiple areas and numerous neurons, such as mesoscopic calcium imaging [147], wide-field two-photon imaging [148], and multi-site two-photon imaging [149], may also be useful for this direction of research.
2. New technologies to monitor neural activity during social behavior enormously increased our knowledge of the underlying mechanisms within a single individual. An extension of such techniques to performing simultaneous recording of brain dynamics from two or more interacting animals opens a new avenue for exploring inter-brain neural dynamics that may serve as neural correlates for shared social variables [150]. Recent work in bats and mice reveals inter-brain synchrony in the PFC during social interaction [151, 152]. How do such inter-brain dynamics emerge from the action of specific neural circuits? How are they implicated in social defects in neuropsychiatric disorders? And how do inter-brain dynamics during interaction in the real world relate to those in physical-digital social interaction (Fig. 4)? For example, the “mouse metaverse”, the amalgamation of virtual reality (VR) and physical reality that can provide an immersive three-dimensional social experience in a digital space [153, 154], offers opportunities for studying novel aspects of social communication in mice. What are the inter-brain dynamics of mice like when they interact with each other via avatars of themselves in an immersive virtual environment? Combined with the use of empathy and prosocial behavior, we may be able to visualize the “mouse mind”.

**Fig. 4:**
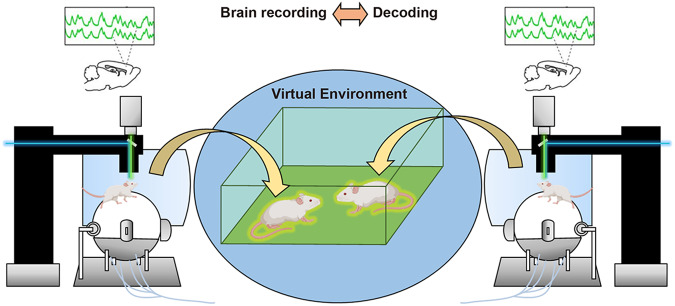
Social interaction in mouse “metaverse”. Synchronized dual microscopes connected via a shared virtual reality allow researchers to study neural dynamics and circuit mechanisms underlying social encounters and interactions of mice in a virtual world.
3. To take full advantage of our knowledge about the circuit mechanisms of social behavior, it is important to pursue not only pharmaceutical interventions but also non-pharmaceutical therapies. How can we establish circuit-based cures for disorders that affect social functioning? Despite its invasiveness, deep brain stimulation through implanted electrodes that directly intervene in pathological neural circuits has been applied to treat various neurological and neuropsychiatric disorders [155]. For the ASD population, non-invasive brain stimulation techniques such as transcranial magnetic stimulation and transcranial direct current stimulation have been used for treatment and rehabilitation, and meta-analyses of studies identify some beneficial effects on symptoms, including the social domain [156–158]. However, the efficacy, specificity, and safety of the current methods still face technical challenges. Next-generation non-invasive or minimally invasive neuromodulation technologies that use electric, optical, magnetic, and acoustic signals may offer a prospect for the clinical applications of circuit-specific brain stimulation therapy [159].

Humans can perform the most complex level of social behavior, and the action of brain networks that support such outstanding quality is extraordinarily intricate. Insights gained from future research using rodents as a model and their extension to explore principles shared with humans will continue to advance our understanding of the biological underpinnings of social behavior and associated deleterious changes. This endeavor will eventually lead to the development of circuit-based therapies for relevant disorders.
